# Modification of polyamide TFC nanofiltration membrane for improving separation and antifouling properties

**DOI:** 10.1039/c8ra01374h

**Published:** 2018-04-20

**Authors:** Li-Fen Liu, Xiang Huang, Xiao Zhang, Ke Li, Yan-Li Ji, Chun-yang Yu, Cong-Jie Gao

**Affiliations:** Center for Membrane and Water Science and Technology, Ocean College, Zhejiang University of Technology Hangzhou 310014 China lifenliu@zjut.edu.cn; College of Chemical Engineering, Zhejiang University of Technology Hangzhou 310014 China; Collaborative Innovation Center of Membrane Separation and Water Treatment of Zhejiang Province Hangzhou 310014 China; School of Chemistry & Chemical Engineering, State Key Laboratory of Metal Matrix Composites, Shanghai Jiao Tong University 800 Dongchuan Road Shanghai China 200240

## Abstract

In this work, a dendrimer trimesoyl amide amine (TMAAM) monomer was proposed to be used as a key functional monomer to modify the conventional aromatic polyamide thin-film composite (TFC) nanofiltration (NF) membrane, and a new kind of TMAAM-based semi-aromatic polyamide composite NF membrane was thus prepared by interfacial polymerization. The effects of the PIP/TMAAM ratio (PIP = piperazine) on the membrane chemical structure, surface properties and separation performances were investigated systematically. With the increase in TMAAM content loaded in the membrane, the water flux strongly increased but the salt rejection decreased only slightly. When the PIP/TMAAM ratio was 1, the membrane NF-2 exhibited a smoother and more hydrophilic surface, as a result of which it displayed an optimum separation performance for different valent salts. In addition, the TMAAM modified TFC membrane presented an extremely high rejection to negatively charged dye molecules and high permeation for monovalent salts, leading to good prospects for dye/salt separation application. Moreover, both the water flux and salt rejection of the TMAAM-based membrane were stable in a long-term running process, and the membrane showed a favourable anti-fouling property and efficient cleaning recovery. Therefore, this work provides a new type of semi-aromatic polyamide composite NF membrane fabricated by a facile and straightforward method *via* interfacial polymerization with high hydrophilicity, good stability and strong anti-fouling property.

## Abstract 2

TMAAM, a new dendrimer, was used to modify the conventional aromatic polyamide NF membrane *via* interfacial polymerization method, and the result TMAAM-based semi-aromatic polyamide NF membrane strong dye rejection, favourable antifouling property and good stability.

## Graphical abstract

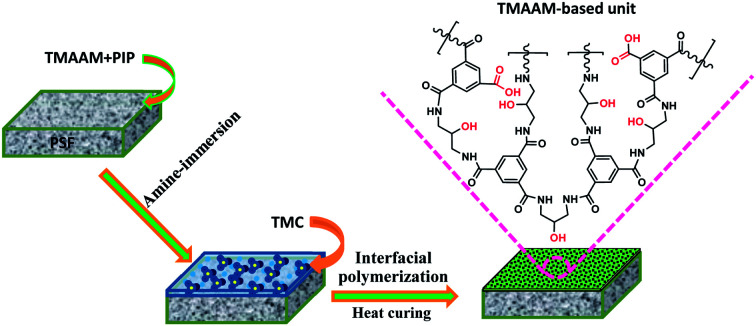

## 1. Introduction

Membrane separation is a new field of technology, including reverse osmosis, nanofiltration, ultrafiltration, electrodialysis, membrane bioreactors, and so on.^1–4^ Membrane separation offers many advantages, *e.g.* high efficiency, energy saving, environmental protection, simple equipment and easy operation *etc.*, and has been widely used in water treatment, highly efficient substance separation, energy saving and environmental remediation.^5–8^ Because the traditional reverse osmosis is characterized by high operating pressure and large energy consumption, nanofiltration, with relatively low operating pressure and high permeation flux, has emerged as an alternative and has become a research hotspot in the field of membrane separation.^9–12^

Nanofiltration (NF) is a pressure-driven membrane separation process. The pore size of an NF membrane (about 1 nm) is intermediate between an ultrafiltration membrane and a reverse osmosis membrane. Based on both the steric hindrance effect and electrostatic repulsion effect, NF membranes can be used to separate different valent salts and various organic molecules (molecular weight cut-off [MWCO] in the range 200–1000). Nowadays, NF is applied in the production of drinking water, recovery and removal of small organic compounds, treatment of industrial waste water, separation and purification of biologicals and pharmaceuticals, and the extraction and refining of food and petroleum substances, and so on.^13–15^ The NF membrane is the core of NF technology. In order to meet the requirements of the complex systems used in practical applications, it is necessary to develop excellent NF membranes with high perm-selectivity, good stability and anti-fouling properties.

NF membranes are normally divided into organic polymeric membranes, inorganic membranes and organic-inorganic hybrid membranes. In the current membrane market, the most widely commercially available membranes are polymeric. However, their water flux and anti-fouling performances require further improvement.^16–18^ A significant amount of research has been carried out to improve membrane performance, for example, developing new types of membrane materials,^22^ modifying membrane surfaces,^19^ as well as incorporating nanomaterials into membranes.^20,21^ Designing and synthesizing new functional monomers is a widespread and effective approach for improving the performance of NF membranes. Jin *et al.*^23^ selected 2,2-oxydiethylamine as the functional monomer, and fabricated polyamide membranes with piperazine (PIP) and trimesoyl chloride (TMC) by interfacial polymerization. The resultant modified membranes exhibited high water flux (35.6 L m^−2^ h^−1^) and good anti-fouling performance. Ang *et al.*^24^ prepared a series of thin-film composite polyamide NF membranes by incorporating different monoamines, *e.g.* 4-aminobenzoic acid (ABA), 6-aminocaproic acid (ACA), and 3-aminopropanoic acid (APA). The ABA modified polyamide NF membrane showed an enhanced perm-selectivity and improved anti-fouling property. Recently, Kong *et al.*^25^ introduced hyperbranched polyester (HPE) into PIP aqueous solution, and then reacted this with TMC in organic solution *via* interfacial polymerization on an ultrafiltration support surface. The water flux of the obtained membrane was increased by nearly 60% due to the looser membrane structure derived from incorporation of HPE into the membrane.

**Fig. 1:**
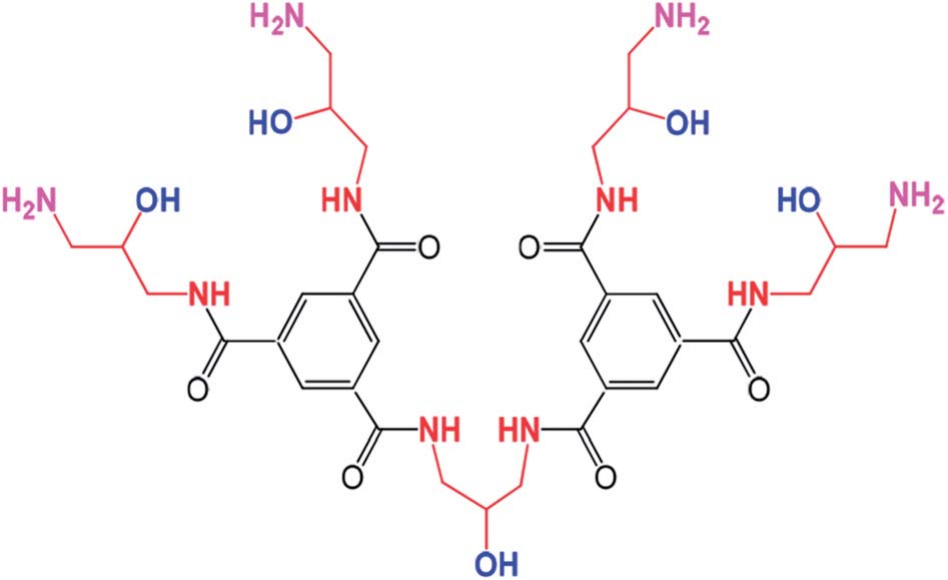
Molecular structure of TMAAM.

In recent years, we have successfully synthesized a new type of dendrimer, trimesoyl amide amine (TMAAM, Fig. 1),^26,27^ which comprises multiple hydroxyl groups and aliphatic amines. It is hypothesized that the hydrophilicity and anti-fouling properties of membranes would be significantly improved *via* incorporating TMAAM. In this study, TMAAM was combined with PIP as the key functional monomer to react with TMC through interfacial polymerization on a polysulfone (PSF) ultrafiltration support membrane, by which a new type of semi-aromatic polyamide composite NF membrane was obtained. Due to the existence of a three-dimensional dendritic structure and multiple hydroxyl groups, water channels and/or aggregate pores could be formed within the TMAAM-based TFC NF membranes, which improved both the water flux and fouling resistance of the resultant membranes.

## 2. Experimental section

### 2.1 Materials and reagents

A PSF ultrafiltration support membrane with a MWCO around 20 000 g mol^−1^ was purchased from Hunan Keensen, China. Monomers including TMC (>99.0%) and PIP (>99.5%) for interfacial polymerization were purchased from J&K Products Catalog. Methyl blue, semixylenol orange, safranine T and neutral red, used as the dye molecules to evaluate the separation performance of the membranes, were purchased from MACKLIN. Analytical standard bovine serum albumin (BSA) was purchased from Shanghai Aladdin Reagent Company and used as a model foulant. All other chemicals were analytical reagents and used directly without further purification.

### 2.2 Preparation of TFC nanofiltration membrane

**Fig. 2:**
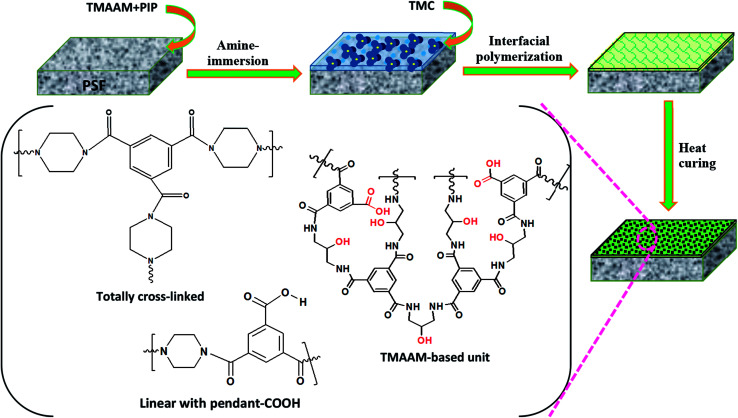
Schematic diagram of the preparation of TMAAM modified TFC membranes and their chemical structure.

Pristine and TMAAM modified TFC NF membranes were prepared by interfacial polymerization (Fig. 2). In this study, the concentration of TMC in *n*-hexane was fixed at 0.15 wt%, while the compositions of the aqueous phase solutions containing different concentrations of TMAAM and PIP are listed in Table 1. First, the PSF support was immersed in the PIP/TMAAM aqueous solution for 2 min, and then excess aqueous solution was drained off from the membrane surface and air-dried at ambient temperature until no liquids remained. Subsequently, the membrane was placed into contact with the organic solution to induce interfacial polymerization for 40 s, and then a polyamide thin film was found to have formed on the surface of the PSF-ultrafiltration membrane after removing excess organic phase solution. The resultant membranes were heated for 5 min in an oven at 60 °C for further polymerization. Finally, the membranes were stored in de-ionized (DI) water before examination. The resultant membranes prepared from different weight ratios of TMAAM/PIP were named as NF-*x* (*x* = 0, 1, 2, 3) respectively.

**Table 1:** Compositions of aqueous phase solution during the interfacial polymerization

No.	PIP%	TMAAM%	SDS%
NF-0	1	0	0.15
NF-1	0.75	0.25	0.15
NF-2	0.5	0.5	0.15
N F-3	0.25	0.75	0.15

### 2.3 Membrane characterization

The membrane surface chemical structures and compositions were analyzed using attenuated total reflectance Fourier transform infrared spectroscopy (ATR-FTIR Nicolet is50) and X-ray photoelectron spectroscopy (XPS, Kratos AXIS Ultra DLD, Shimadzu-KRATOS, Japan). The surface morphologies of the membranes were observed using field emission scanning electron microscopy (SEM, Hitachi SU8010, Japan) and atomic force microscopy (AFM, Bruker, Dimension Icon, USA). Before SEM observation, the membrane samples were sputtered with gold. The membrane surface roughness (Ra) was measured in air atmosphere at room temperature by AFM. Ra was calculated from the height profile of each 5.0 μm × 5.0 μm three-dimensional AFM image. The sessile drop method was adopted to measure the static contact angles of DI water on the dried surface of the membrane at 25.0 °C by employing a contact angle meter (Dataphysics OCA50AF, Germany). At least 5 measurements on different locations of each membrane sample were performed and averaged to obtain the contact angle of the measured membrane. The membrane surface streaming potential was measured with an electrokinetic analyzer (EKA, Anton Paar SurPASS 3, Austria) using 0.001 mol L^−1^ KCl aqueous solution at 25.0 °C and pH = 6.5. The surface zeta potential was then determined from the measured streaming potential according to the Helmholtz–Smoluchowski equation; the data presented are average values from five samples of each membrane type.

### 2.4 Evaluation of membrane water flux and solute rejection

Membrane water flux and solute rejection were evaluated through standard cross-flow permeation tests employing a laboratory scale filtration setup with three parallel circular filtration cells. All the permeation tests were conducted under the constant temperature of 25.0 °C, *trans*-membrane pressure of 6.0 bar, and pH of 6.5 ± 0.2. Circular membrane samples with an effective filtration area of 19.6 cm^2^ loaded in the stainless filtration cells were pressurized at 7.0 bar with DI water for 1 h before the permeation test to ensure stable membrane flux. The membrane water flux (J, L m^−2^ h^−1^) was determined by the following eqn (1):1
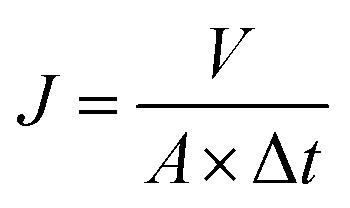
where *A* (m^2^) is the effective area of the membrane for permeation, and *V* (L) is the volume of permeated water over a time interval Δ*t* (h).

The membrane solute rejection performance was determined with single-solute aqueous solutions including Na_2_SO_4_, MgSO_4_, MgCl_2_ and NaCl, or with PEG samples having different molecular weights, or with the dyes methyl blue, semixylenol orange, safranine T and neutral red. The concentrations of the dyes, PEG and inorganic salts were 100, 100 and 1000 mg L^−1^, respectively. The observed solute rejection *R* (%) was calculated according to the following eqn (2):2
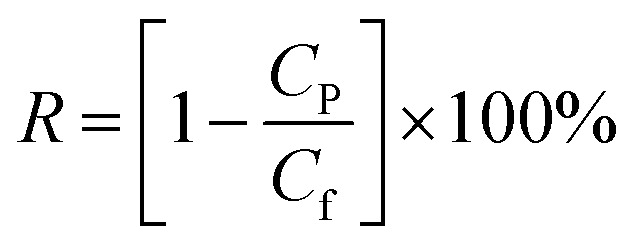
where *R* is the solute rejection, and *C*_p_ and *C*_f_ (g L^−1^) are the concentrations of solute in the permeate and feed, respectively. The salt concentration in the feed and permeate was calculated according to the electrical conductivity of the corresponding salt solution using an electrical conductivity meter (DDS-11A, Hangzhou Dongxing Instrument Co., China). MWCO was defined as the molecular weight of PEG for which the rejection of the membrane was about 90%, and the PEG concentration was measured by a total organic carbon analyzer (TOC-V CPN, Shimadzu, Japan), while the dye concentration was measured using an ultraviolet-visible spectrophotometer (TU-1810PC, Beijing) at the maximal absorption wave-length of each organic dye.

### 2.5 Evaluation of membrane anti-fouling property

BSA was used as the model foulant to evaluate the anti-fouling property of the resultant NF membranes. The flux decline ratio (FDR) and water recovery ratio (FRR) were determined through the following eqn (3) and (4),3
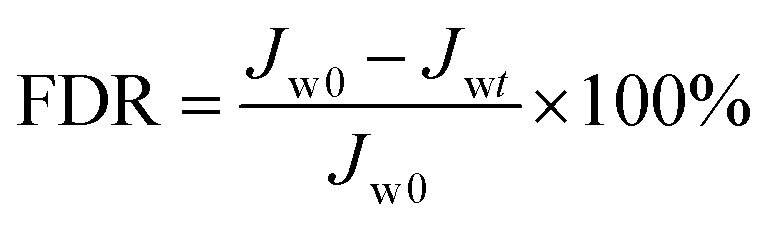
4
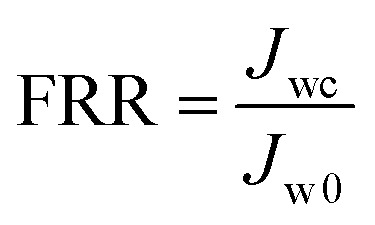
where *J*_w0_, *J*_wt_ and *J*_wc_ are the water fluxes of the original, filtrated and water-cleaned membranes. A lower FDR value means a better antifouling property, while a higher FRR value indicates a higher cleaning efficiency.

## 3. Results and discussion

### 3.1 Chemical composition and structures of TFC membranes

**Fig. 3:**
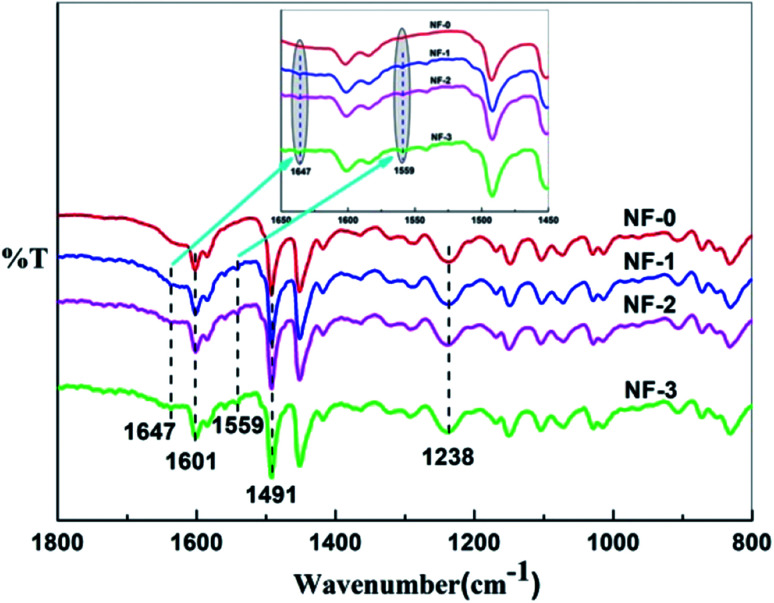
ATR-FTIR spectra of the TFC NF-*x* (*x* = 0–3) membranes.

The chemical compositions of pristine TFC membrane and TMAAM modified TFC membranes were examined by ATR-FTIR. As shown in Fig. 3, the characteristic peaks at 1601 and 1491 cm^−1^ corresponding to the carbon–carbon double bond of the benzene ring were observed for all of the TFC membranes. A peak at 1238 cm^−1^ was seen on the ATR-FTIR spectrum of NF-0, which was attributed to the C–N stretching vibration of imide groups in the polyamide membrane. Compared with the pristine TFC membrane, new absorption peaks at 1647 cm^−1^ and 1559 cm^−1^, ascribed to the stretching vibration of C=O and the bending vibration of N–H in amide groups, were visualized in the spectra of NF-1, NF-2 and NF-3. These characteristic peaks of amide groups were all derived from the semi-aromatic amide bond in the TMAAM unit of the modified TFC membranes, while the secondary amine groups had reacted with TMC, so that there was no N–H bending vibration peak in the ATR-FTIR spectrum of pristine TFC membrane. These results demonstrated that the modified TFC membranes had been successfully fabricated with PIP, TMC and TMAAM. The chemical structure of the membranes was further investigated with XPS.

XPS with a radiant depth of a few nanometres is normally used for probing the chemical structure of a thin-film layer. In this work, the quantitative elemental composition of the selective layer of the composite polyamide membranes was evaluated by XPS. In general, a typical polyamide selective layer is usually composed of a mixture of cross-linked and linear polyamide chemical structures. The elemental composition and elemental ratio of the TMAAM monomer, the pristine TFC membrane and modified TFC membranes are shown in Table 2. The polyamide selective layers of the modified TFC NF membranes were prepared with PIP, TMAAM and TMC, thus they mainly consist of three elements, *i.e.* C, N, and O (Table 2). The oxygen content primarily comes from the C=O part of the amide bonds, the –OH in TMAAM and the –COOH produced by hydrolysis of the unreacted acyl chloride groups. The nitrogen content is from the C–N part of the amide bonds. As shown in Table 2, the ratio of O/N is closely related to the cross-linking degree of the polyamide structure, wherein, the higher the cross-linking degree of the polyamide layer, the lower the O/N ratio. The O/N ratio of pristine TFC membrane NF-0 is 1.43, which is between the ideal linear and fully cross-linked structures, but much closer to the latter one. This indicates that there are more cross-linked structures in the NF-0 membrane, which confirms the existence of amide bonds in the membrane. Moreover, it is worth noting that the ratio of O/N in TMAAM is between the values of ideal cross-linked and linear structures of PIP-TMC polyamide. Upon introducing a small amount of TMAAM segments into the polyamide membrane, the O/N ratio slightly decreases. Furthermore, the O/N ratio of the modified TFC membranes increases from 1.37 to 1.90 with increasing TMAAM concentration. All of the above results demonstrate that the TMAAM was incorporated into the membrane, and the cross-linking degree of the modified TFC membrane was reduced to some extent.

**Table 2:** Elemental compositions of the TFC membranes from ideal structures and practical XPS measurement

Samples	Atomic composition from XPS			
	C%	N%	O%	O/N
TMAAM	61.46	17.07	21.46	1.26
PIP-TMC				
Ideal linear structure	62.90	11.29	25.81	2.29
Ideal cross-linking structure	66.67	15.56	17.78	1.14
NF-0	72.93	11.15	15.92	1.43
NF-1	71.89	11.86	16.24	1.37
NF-2	71.53	11.14	17.33	1.55
NF-3	74.98	8.61	16.40	1.90

### 3.2 Surface morphologies of TFC membranes

**Fig. 4:**
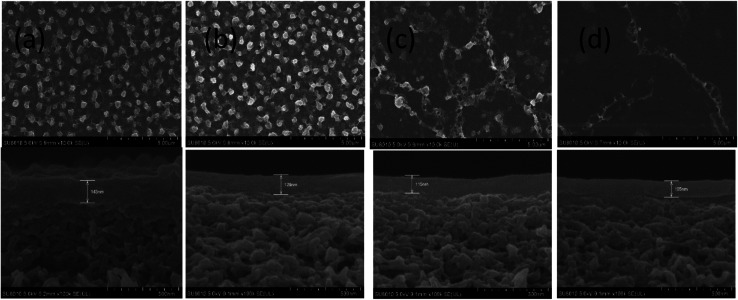
FESEM images of the TFC membranes: NF-0 (a), NF-1 (b), NF-2 (c) and NF-3 (d).

SEM and AFM micrographs were recorded to observe the surface morphologies of the pristine polyamide membrane NF-0, and TMAAM modified TFC membranes including NF-1, NF-2, and NF-3 (Fig. 3 and 4). Fig. 3 shows a peak and valley structure for NF-0, which is the typical morphology for an interfacial polymerized thin-film layer. Compared with NF-0, the nodular structure on the TMAAM modified TFC membranes began to deform and latterly disappeared with increasing TMAAM content. This variation is probably due to the difference of the diffusion rate between PIP and TMAAM aqueous monomers during the interfacial polymerization process. It is also worth noting that the polyamide skin layer became thinner with the addition of TMAAM, which was helpful for improving the flux of the membrane (please see Section 3.5). In addition, the AFM results are well consistent with the SEM images, and the average roughness of all the membranes decreases in the following order: NF-0 (74.6 nm) > NF-1 (66.5 nm) > NF-2 (50.1 nm) > NF-3 (38.4 nm). Therefore, the TMAAM-based polyamide membranes are smoother than the pristine polyamide membrane. From the above XPS analysis, it is known that the crosslinking degree of the polyamide membrane decreases and the linear structure of the membrane increases with the increase in TMAAM content of the membrane, thus the membrane's surface becomes much smoother. Generally, a smoother surface is more conducive for improving the anti-fouling performance of the membrane.

### 3.3 Hydrophilicity and charge characteristics of TFC membranes

**Fig. 5:**
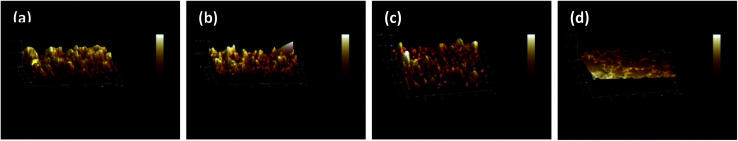
AFM images of the TFC membranes: NF-0 (a), NF-1 (b), NF-2 (c) and NF-3 (d).

**Fig. 6:**
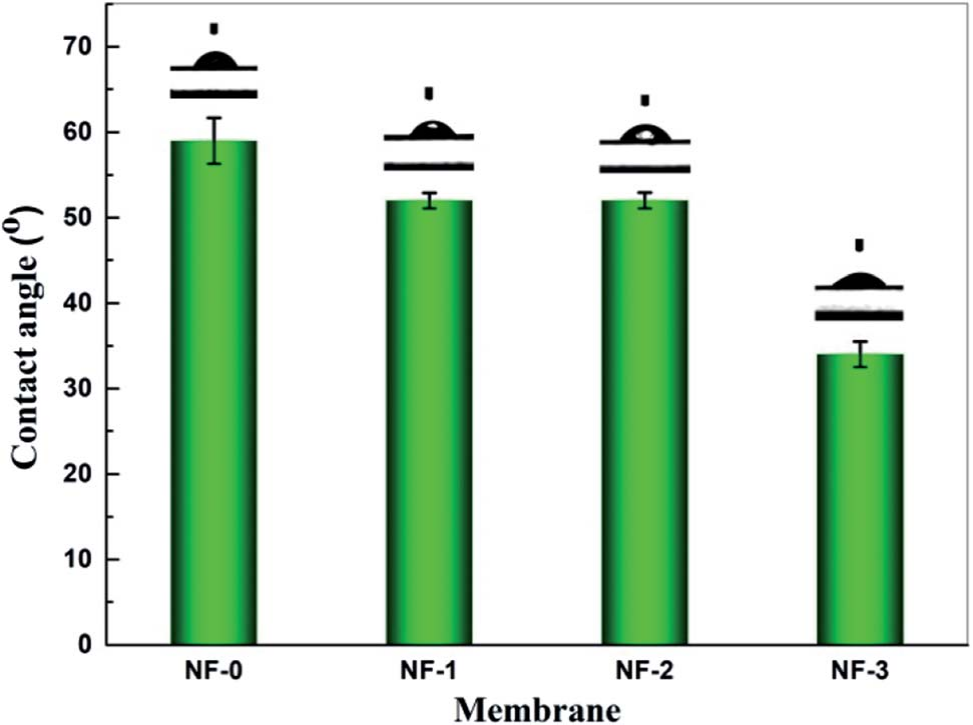
Water contact angle of NF-0, NF-1, NF-2 and NF-3.

The water contact angle was used to evaluate the hydrophilicity of the TFC membranes (Fig. 6). In general, a membrane is hydrophilic if the contact angle *θ* is less than 90°, and hydrophobic if *θ* is more than 90°. The smaller the contact angle is, the higher the membrane hydrophilicity is. As shown in Fig. 5, the water contact angle for the four TFC NF membranes decreases from ∼59° to ∼34° with the increasing TMAAM concentration from 0 to 75 wt%. This indicates that the hydrophilicity of the polyamide surface increases with the increasing TMAAM content in the membrane. This is probably because there are more hydroxyl groups on the membrane surface with the increasing content of TMAAM. Moreover, according to the above XPS analysis, the crosslinking degree of the polyamide layer decreased with the increase of TMAAM content, as a result of which, more unreacted acyl chloride groups were hydrolysed into carboxyl groups, which contributed to the enhancement of the hydrophilicity of the membrane surface. A highly hydrophilic surface is an important characteristic in terms of good membrane permeability and anti-fouling property, which will be discussed in detail later.

**Fig. 7:**
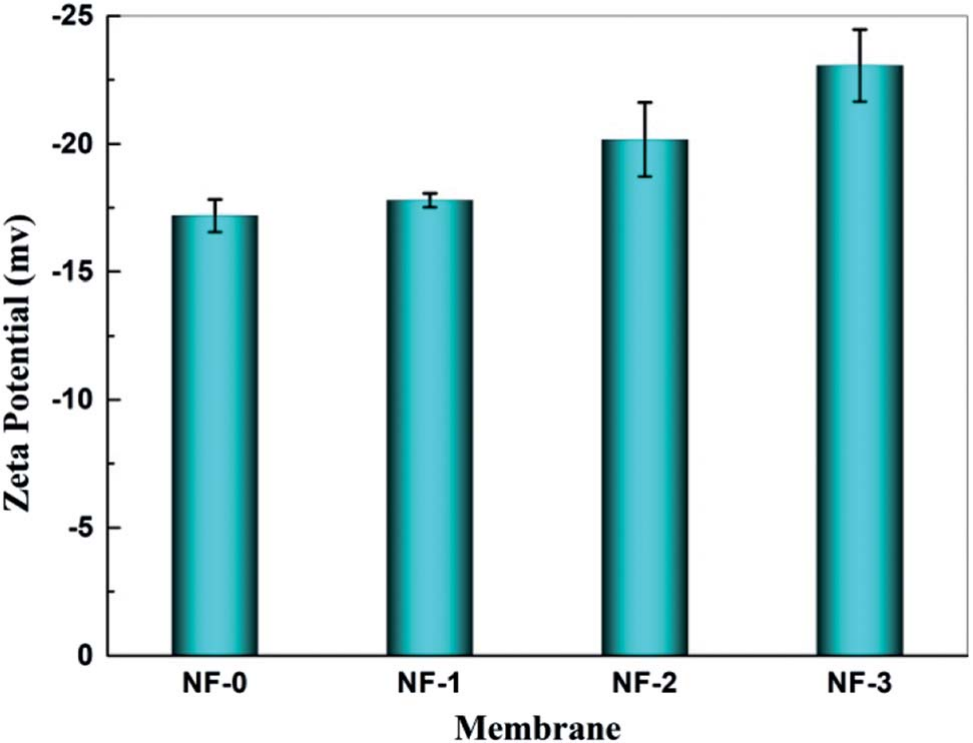
Zeta potential of the membranes NF-0, NF-1, NF-2 and NF-3 tested at 6.0 bar and 25.0 °C, pH = 6.

NF membranes separate substances by both the steric hindrance effect and electrostatic interaction effect, thus the charge characteristics of the membrane surface are very important for NF membranes. Fig. 7 gives the zeta potential of pristine and TMAAM modified TFC NF membranes. The zeta potential of TFC NF membranes varied from ∼−17 mV to ∼−23 mV when the membranes were tested with 0.01 M KCl aqueous solution at pH 6.5. It was found that the isoelectric points of both pristine TFC membrane and TMAAM modified TFC membranes were at about pH 4.0. When the pH value of the aqueous solution is lower than 4.0, more protons adsorb on the amide groups, thus the membranes are positively charged. However, the membrane surface would become more negative in aqueous solution with pH higher than 4.0. This is because the hydrolysis of unreacted acyl chloride groups generates more carboxyl groups, while more hydroxide ions are adsorbed on the membrane surface in basic aqueous solution. The negative charge density of the TMAAM modified TFC membrane is higher than the pristine TFC membrane, *i.e.*, there are more carboxyl groups on the TMAAM-based membrane surface. This indicates that the polyamide layer of the TMAAM modified TFC membranes has a lower crosslinking degree and more linear structure, which is consistent with the result of XPS analysis.

### 3.4 Molecular weight cut-off of TFC membranes

**Fig. 8:**
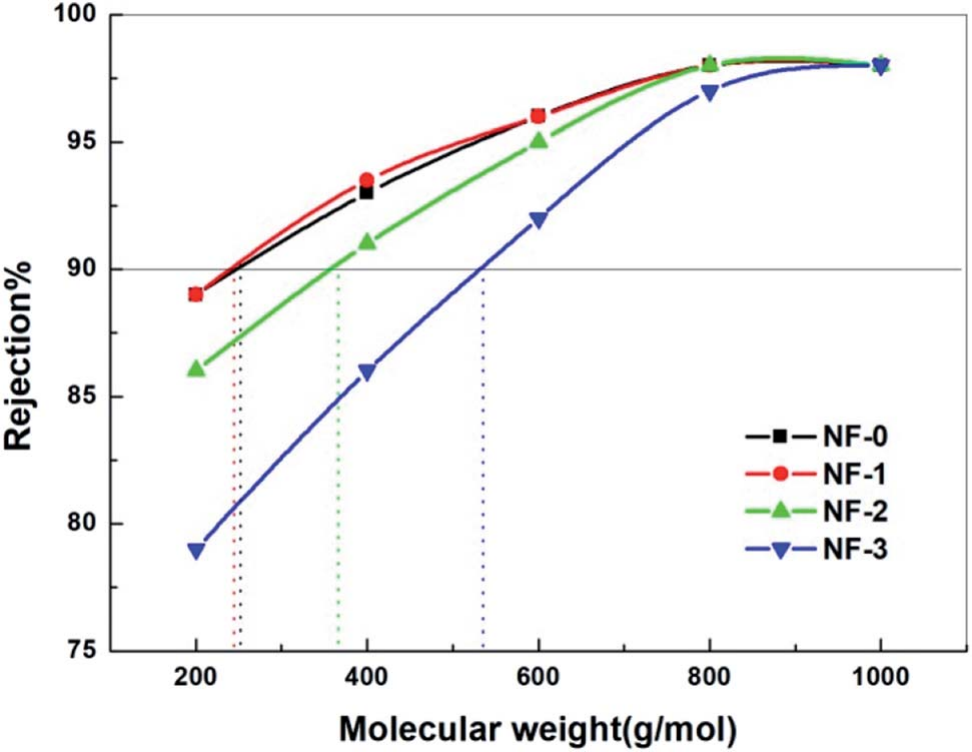
Rejection of the NF-*x* (*x* = 0, 1, 2, 3) membranes to PEGs with different molecular weights tested with 100 mg L^−1^ feed aqueous solution at 6.0 bar and 25.0 °C.

Apart from the membrane surface properties, the NF performance of TFC membranes is also associated with the membrane MWCO value. The MWCO value of the TFC NF membranes was measured by using neutral solute PEGs with different molecular weights. The obtained MWCO value is the molecular weight of PEG corresponding to the 90% rejection of the membrane. As presented in Fig. 8, the MWCO value of the TFC membranes increases from 246 Da to 462 Da with the increasing TMAAM concentration. This indicates that the chemical cross-linking degree of the TFC NF membranes decreases with the increasing TMAAM concentration, which is consistent with the XPS analysis results. This is because the branched molecule TMAAM incorporated in the polyamide layer leads to a looser polyamide structure, which allows larger PEG molecules to be easily transported through the membrane, resulting in a higher MWCO value for the TMAAM-based TFC NF membranes.

### 3.5 Separation performance of TFC membranes

**Fig. 9:**
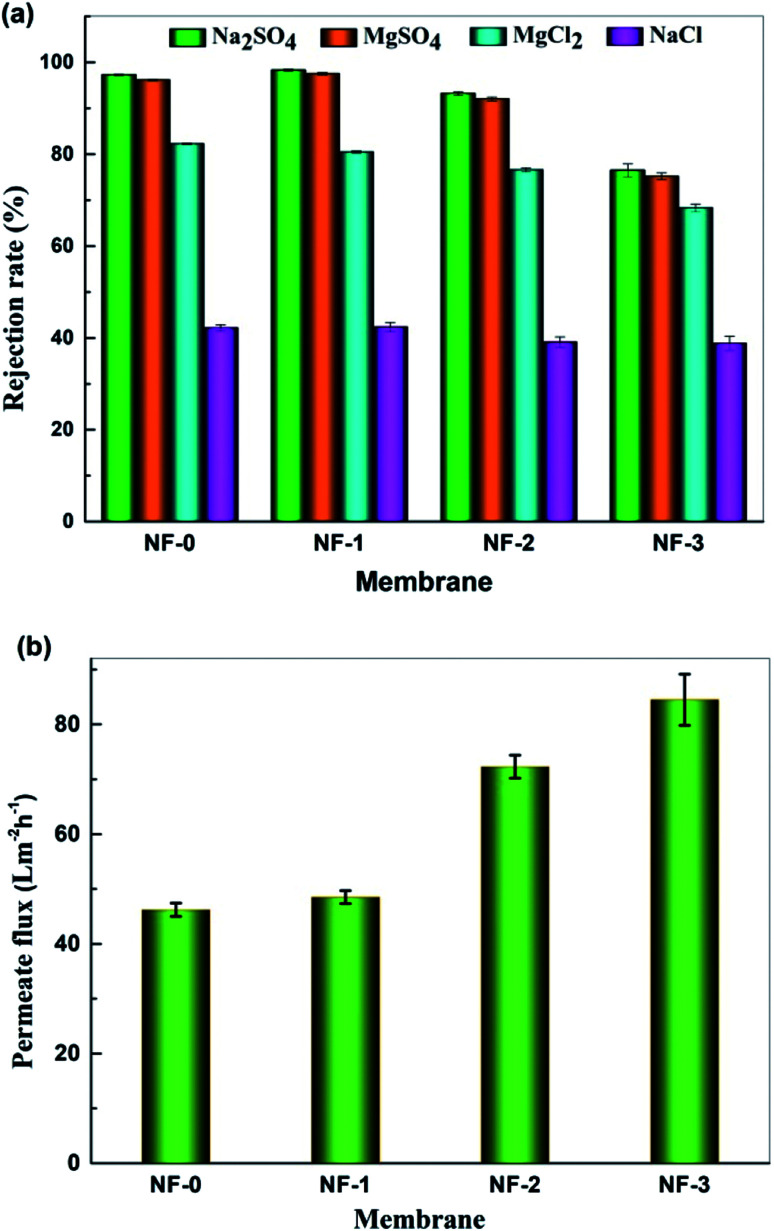
Separation performance of the prepared TFC NF-*x* (*x* = 0, 1, 2, 3) membranes: (a) Rejection of different salts and (b) pure water flux tested with 1000 mg L^−1^ feed aqueous solution at 6.0 bar and 25.0 °C.

The separation performance of the TFC NF membranes was investigated with 1000 mg L^−1^ salt aqueous solution at 25 °C and 6 bar. Fig. 9a shows the salt rejection of the TFC NF membranes, and it can be seen that the salt rejection of pristine TFC membrane to Na_2_SO_4_, MgSO_4_, MgCl_2_ and NaCl is 97.3%, 96.2%, 82.3% and 42.2%, respectively. It is well known that NF performance is governed by both Donnan exclusion and size exclusion mechanisms. This variation trend of salt rejection indicates that Donnan exclusion plays an important role in controlling the separation performance of the TFC NF membranes.^28,29^ The zeta potential results in Fig. 7 show that the TFC NF membranes are negatively charged, which means that there is much stronger electrostatic repulsive interaction between divalent anions (SO_4_^2−^) and the membrane surface, thus the rejections for both Na_2_SO_4_ and MgSO_4_ are higher than that for MgCl_2_ and NaCl. Fig. 9b shows that the water flux of the TFC NF membranes dramatically increases when introducing TMAAM into the membranes. The water flux increases from 46.2 L m^−2^ h^−1^ to 84.5 L m^−2^ h^−1^ with the TMAAM concentration increasing from 0 to 75 wt%. When the TMAAM concentration is 50 wt%, the water flux of NF-2 increases up to 72.3 L m^−2^ h^−1^, which is about 56.5% higher than the pristine TFC membrane. Meanwhile, the salt rejection of the membrane changes slightly, and the rejections of NF-2 to Na_2_SO_4_ and NaCl are 92.4% and 31.1% respectively. The increase in water flux is possibly attributed to the higher hydrophilicity of the membrane surface with more hydroxyl groups and carboxyl groups. Moreover, the cross-linking degree of the membrane decreases when further increasing the TMAAM concentration to more than 50%, which reduces the salt rejection. In conclusion, the TFC NF membrane with optimum TMAAM content exhibits high water permeability and salt selectivity, and has the prospect of application in desalination and substance separation.

**Fig. 10:**
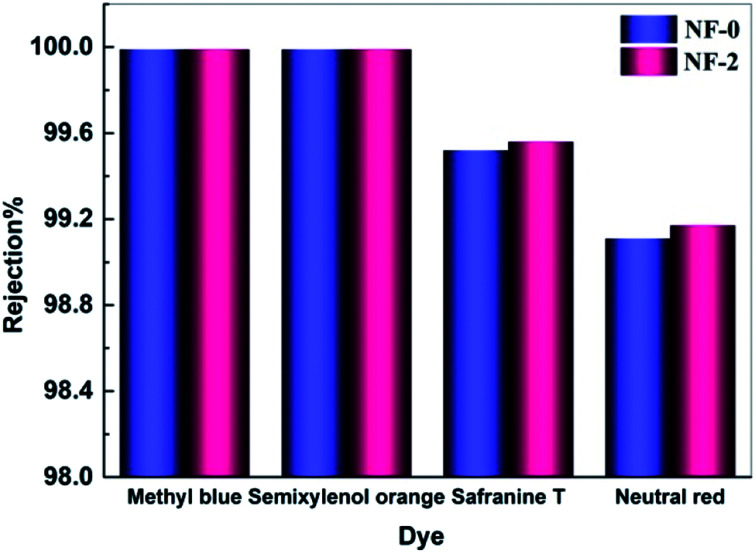
Rejection of NF-2 membrane to different dye molecules tested with 100 mg L^−1^ feed aqueous solution at 6.0 bar and 25.0 °C.

Textile wastewater contains compounds with high chromaticity and salinity, which causes serious pollution and resources-waste after untreated discharge. NF is emerging as a competitive separation technology to resolve this problem. Thus, the TMAAM modified TFC NF membrane with high water permeability and solute selectivity is an alternative candidate to be used for dye/salt separating applications. Fig. 10 shows the separation performance of the TMAAM modified TFC membrane NF-2 and the blank membrane NF-0 to dye molecules of methyl blue (799 Da), semixylenol orange (547 Da), safranine T (350 Da) and neutral red (288 Da). Both the NF-0 and NF-2 rejected the majority of dye molecules (rejection > 99%). It is also notable that NF-2 exhibited higher rejection to safranine T and neutral red than NF-0 while allowing most of the monovalent salt NaCl to permeate (Fig. 9a). This is because the NF-2 membrane with high negative charge density has a much stronger electrostatic repulsive interaction between negatively charged dye molecules and the membrane surface, resulting in a very high dye rejection. Compared with some other polyamide NF membranes reported in the literature, NF-2 also exhibited prominent rejection to methyl blue (Table 3).

**Table 3:** Comparison of the rejection to methyl blue between this work and other studies in the literature

Literature	30	31	32	33	This work
Rejection%	63.7	95.10	98.9	99.8	99.99
Pressure/bar	3	1	2	5	6
*C*_dye_/ppm	500	100	500	50	100

### 3.6 Stability and anti-fouling performance of TFC membranes

**Fig. 11:**
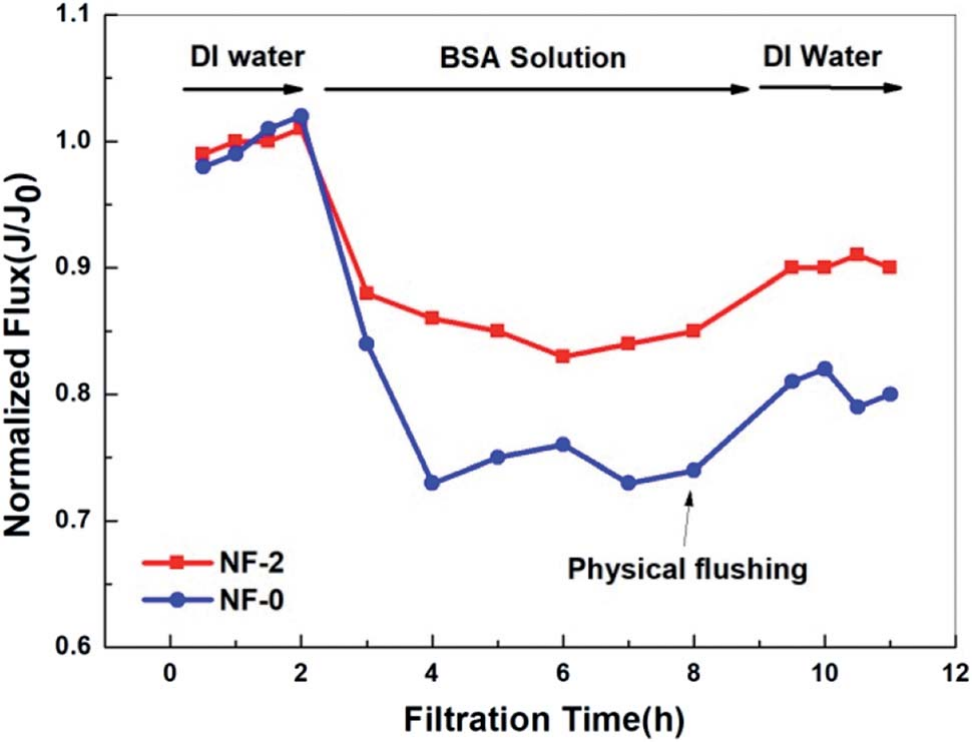
Effect of operation time on flux and solute retention of NF-2 tested with Na_2_SO_4_ 1000 mg L^−1^ aqueous solution at 25 °C and 6 bar.

The stability and anti-fouling property of membranes are vital for NF applications. In this work, a long-term test of the TMAAM modified TFC membrane was conducted with 1000 mg L^−1^ Na_2_SO_4_ aqueous solution at 25 °C and 6 bar (Fig. 11). Both the water flux and salt rejection changed only slightly during the 60 h running process, remaining at about 60 L m^−2^ h^−1^ and 98%, respectively. In actual applications, membranes are easily contaminated by various pollutants, which reduces the membrane separation performance and shortens the service life of the membrane, resulting in a high operating cost. Proteins are a typical class of pollutants in the NF process, thus the fouling resistance of the pristine TFC and TMAAM modified TFC membrane was assessed using BSA as model protein in 500 mg L^−1^ aqueous solution at pH = 7, 25 °C.

**Fig. 12:**
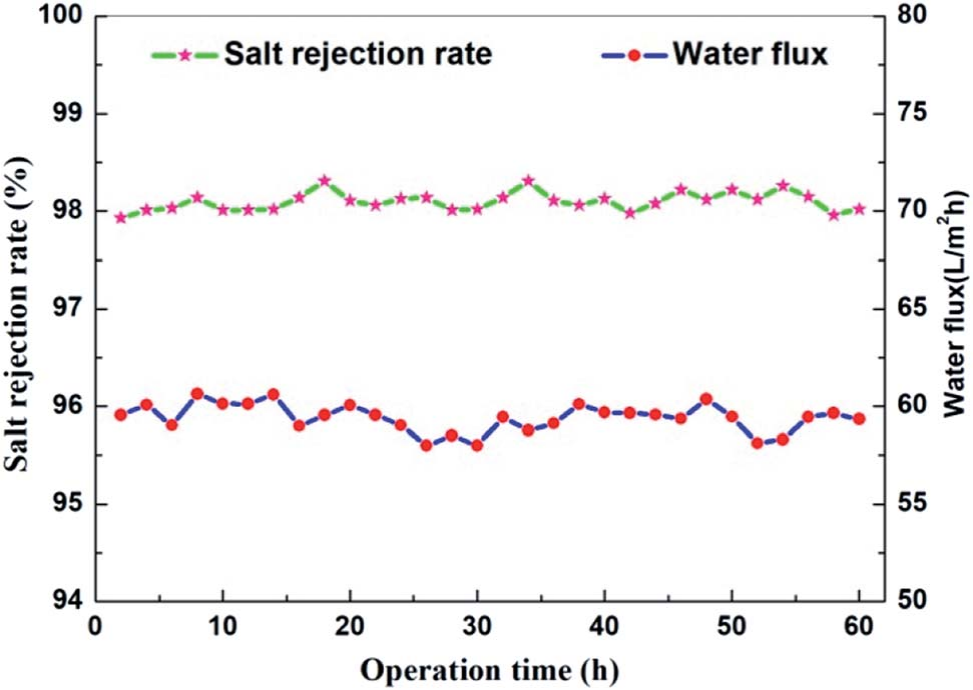
Time-dependent normalized fluxes (*J*_t_/*J*_0_) for TFC membranes NF-0 and NF-2 in filtration of an aqueous solution containing 500 mg L^−1^ BSA (25 °C, pH = 6.5).

As shown in Fig. 12, the water flux decreases much more quickly for NF-0 than for NF-2, and the final time-dependent normalized flux (*J*_t_/*J*_0_) of NF-0 is 27.2% while that of NF-2 is 14.3%. In detail, the flux decline ratio (FDR) and water recovery ratio (FRR) of NF-0 and NF-2 are 14.3%, 27.2% and 81.4%, 90.3%, respectively. Clearly, the fouling resistance and cleaning recovery property are better for the TMAAM modified TFC membrane. This improved anti-fouling property can perhaps be ascribed to the incorporation of TMAAM into the TFC membrane. The TMAAM modified TFC membrane is very hydrophilic, as a result of which there are large amounts of “free water” molecules adsorbed on the membrane surface, which prevent the protein molecules from contacting and firmly adhering on the membrane surface. Moreover, the TMAAM modified TFC NF membrane is negatively charged, and thus has a stronger electrostatic repulsive interaction between the negative BSA (isoelectric point, pI = 4.7) and membrane surface. Thus, the proteins are easily repelled from the smooth membrane surface of the TMAAM modified TFC membrane. In summary, the TFC NF membranes containing TMAAM have an excellent foulant resistance property, making them suitable for substance separation in a range of complex application systems.

## 4. Conclusions

In this work, the dendrimer TMAAM monomer was used to modify a polyamide thin-film composite (TFC) NF membrane, and a new kind of TMAAM-based semi-aromatic polyamide composite NF membrane was thus prepared by interfacial polymerization. The effects of the PIP/TMAAM ratio on the membrane chemical structure, surface properties and separation performances were investigated systematically. With the increasing TMAAM loading content, the water flux of the membrane strongly increases but the salt rejection decreases only slightly. When the PIP/TMAAM ratio is 1, the membrane NF-2, with a smoother and more hydrophilic surface, displays an optimum separation performance for different valent salts. In addition, the TMAAM modified TFC membrane exhibits an extremely high rejection to negatively charged dye molecules and high permeation for monovalent salts, showing good prospects for dye/salt separation application. Moreover, both the water flux and salt rejection are stable in a long-term running process, while the TMAAM modified TFC membrane shows a good anti-fouling property and cleaning recovery efficiency. Therefore, this work provides a new type of semi aromatic polyamide composite NF membrane fabricated by a facile and straightforward method *via* interfacial polymerization with high hydrophilicity, good stability and strong anti-fouling property.

## Conflicts of interest

There are no conflicts to declare.

## Supplementary Material
